# Tobacco Smoke Augments *Porphyromonas gingivalis - Streptococcus gordonii* Biofilm Formation

**DOI:** 10.1371/journal.pone.0027386

**Published:** 2011-11-14

**Authors:** Juhi Bagaitkar, Carlo A. Daep, Carol K. Patel, Diane E. Renaud, Donald R. Demuth, David A. Scott

**Affiliations:** 1 Department of Microbiology and Immunology, University of Louisville, Louisville, Kentucky, United States of America; 2 Center of Oral Health and Systemic Disease, University of Louisville, Louisville, Kentucky, United States of America; East Carolina University School of Medicine, United States of America

## Abstract

Smoking is responsible for the majority of periodontitis cases in the US and smokers are more susceptible than non-smokers to infection by the periodontal pathogen *Porphyromonas gingivalis. P. gingivalis* colonization of the oral cavity is dependent upon its interaction with other plaque bacteria, including *Streptococcus gordonii*. Microarray analysis suggested that exposure of *P. gingivalis* to cigarette smoke extract (CSE) increased the expression of the major fimbrial antigen (FimA), but not the minor fimbrial antigen (Mfa1). Therefore, we hypothesized that CSE promotes *P. gingivalis-S. gordonii* biofilm formation in a FimA-dependent manner. FimA total protein and cell surface expression were increased upon exposure to CSE whereas Mfa1 was unaffected. CSE exposure did not induce *P. gingivalis* auto-aggregation but did promote dual species biofilm formation, monitored by microcolony numbers and depth (both, p<0.05). Interestingly, *P. gingivalis* biofilms grown in the presence of CSE exhibited a lower pro-inflammatory capacity (TNF-α, IL-6) than control biofilms (both, p<0.01). CSE-exposed *P. gingivalis* bound more strongly to immobilized rGAPDH, the cognate FimA ligand on *S. gordonii*, than control biofilms (p<0.001) and did so in a dose-dependent manner. Nevertheless, a peptide representing the Mfa1 binding site on *S. gordonii,* SspB, completely inhibited dual species biofilm formation. Thus, CSE likely augments *P. gingivalis* biofilm formation by increasing FimA avidity which, in turn, supports initial interspecies interactions and promotes subsequent high affinity Mfa1-SspB interactions driving biofilm growth. CSE induction of *P. gingivalis* biofilms of limited pro-inflammatory potential may explain the increased persistence of this pathogen in smokers. These findings may also be relevant to other biofilm-induced infectious diseases and conditions.

## Introduction

Periodontitis is an infectious, chronic inflammatory disease of the supportive structures of the teeth. Smokers often exhibit periodontal disease that is more severe than in non-smokers, with increased alveolar bone loss [1], attachment loss [2], tooth mobility, and tooth loss [3] all apparent and odds ratios of 3 to 7 commonly reported [4]. Moreover, smokers are more likely to be refractory to treatment than non-smokers [5]. Indeed, smoking has been reported to be responsible for more than half of the 15 million periodontitis cases in the US, with 42% and 11% attributable to current and former smoking, respectively [6]. The resultant health burden consumes > $14 billion per annum [7].

Dental plaque in healthy individuals is predominantly comprised of Gram positive commensals, with oral streptococci such as *Streptococcus gordonii* estimated to represent up to 70% of the total bacterial population [8], . However, in periodontitis there is a bacterial succession to a microflora rich in Gram negative, obligate anaerobes that is responsible for the onset and progression of disease [8], [9]. Multiple studies have shown that smokers are more likely to be infected with the key etiological agent of periodontitis, *P. gingivalis*, to harbor higher numbers of *P. gingivalis*, and to exhibit more persistent infection by this pathogen [11], , relative to non-smokers.

*P. gingivalis* persistence in the oral cavity of smokers can be attributed to a compromised immune response and/or increased bacterial virulence. We have recently shown, for example, that *P. gingivalis* adapts to the environmental stress presented by cigarette smoke extract (CSE) by altering the expression of several genes and outer membrane proteins that are key in lowering its inflammatory potential. Interestingly, specific genes (PG2133 and PG2134) in the operon coding for the synthesis and assembly of the major fimbrial antigen of *P. gingivalis* (*fimA*) were induced on exposure to CSE, while several genes in the capsular biosynthesis locus (*capK*, PG0117, PG0118 and *wecC*) were suppressed [15].

During the development of dental plaque, primary colonizers first bind to the salivary pellicle on the tooth surface and provide an attachment stratum that facilitates bacterial succession through multimodal, receptor-ligand mediated, co-adhesive interactions. In order for *P. gingivalis* to establish an oral infection, interactions with primary or early colonizers, such as *S. gordonii*, are critical [16], [17]. For example, the long fimbriae (FimA) of *P. gingivalis* bind to *S. gordonii* glyceraldehyde-3 phosphate dehydrogenase (GAPDH) [17], [18] while the shorter fimbriae (Mfa1) bind to streptococcal surface protein B (SspB) protein [19]. We have previously shown that an 80 amino acid sequence on SspB is critical for Mfa1 adhesion, with a synthetic peptide of this SspB region, named BAR (SspB Adherence Region), a potent inhibitor of Mfa1-dependent *P. gingivalis* - *S. gordonii* biofilms formation [20]. On the other hand, *P. gingivalis* capsule production has been shown to be inversely related to biofilm growth [21].

As CSE-exposure down-regulates capsular genes and upregulates *fimA*-related gene activity, but not Mfa1 activity, we hypothesized that CSE exposure would augment *P. gingivalis* - *S. gordonii* biofilms formation via a FimA-dependent mechanism. We set out to test this hypothesis in a dual species, open flow biofilm model.

## Materials and Methods

### Materials

*P. gingivalis* 33277 and *Streptococcus gordonii* DL1 were purchased from the American Type Culture Collection (Manassas, VA). *Escherichia coli* TOP10, pBAD gIIIA expression plasmid and Platinum PCR Supermix High Fidelity were from Invitrogen (Carlsbad, CA). Gifu Anaerobe Medium [GAM] came from Nissui Pharmaceutical (Tokyo), while brain heart infusion (BHI) media and yeast extract were purchased from Beckton Dickinson (Sparks, MD). HRP-linked anti-Rabbit IgG was from Cell Signaling Technology (Beverly, MA), while anti-FimA and anti-Mfa1 specific antibodies were custom generated by Cocalico Biologicals (Reamstown, PA). Alexa Fluor 488 protein labeling kit to tag anti-*P. gingivalis* antibodies as well as hexidium iodide were purchased from Invitrogen. Tetramethylbenzidine, Manostat Carter 4/8 cassette peristaltic pump, 15 mm×40 mm cover glass, 0.89 mm diameter, platinum-cured silicone tubing and Falcon PVC plates were all purchased from Fisher Scientific (Suwanee, GA). HiTrap Chelating HP affinity columns came from Amersham Biosciences Corp. (Piscataway, NJ). FC71 flow cells were obtained from Biosurface Technologies Corp. (Bozeman, MT). Cloning primers were bought from Biosynthesis (Lewisville, TX) with initial TA-cloning performed using pGEM-T Easy from Promega (Madison, WI) followed by cloning into the pBAD gIIIA expression plasmid from Invitrogen. BAR peptide was synthesized by Biosynthesis Inc. (Lewisville, TX). Standard 3R4F reference cigarettes were obtained from Kentucky Tobacco Research and Development Center (Lexington, KY). West Pico chemiluminescent substrate kits and bacterial protein extraction reagent (B-PER) were purchased from Thermo Scientific (Rockford, IL). α-Bungarotoxin, BSA and 3,3′-Diaminobenzidine (DAB) substrate were purchased from Sigma Aldrich (St. Louis MO). Nitrocellulose membranes came from BioRad (Hercules, CA). TNF-α and IL-6 ELISA kits were purchased from eBioscience (San Diego, CA). Hydroxyapatite discs were purchased from Clarkson Chromatography Products (South Williamsport, PA).

### Bacterial culture and *in vitro* modeling of tobacco exposure

*S. gordonii* DL-1 was cultured in BHI supplemented with 0.5% yeast extract under aerobic conditions, without shaking, at 37°C. *P. gingivalis* 33277 was grown in GAM or GAM conditioned with CSE (GAM-CSE) under anaerobic conditions (86% N_2_, 4% H_2_, 10% CO_2_) at 37°C in a Coy Laboratories (Grass Lake, MI) anaerobic chamber. Bacteria were harvested at mid- to late-exponential phase (O.D. of 1 corresponds to 10^9^ cells ml^-1^). To prepare CSE, cigarette smoke was drawn through 50 ml GAM by using a three-way stopcock and a syringe, with 40 ml ‘drags’ performed over a period of 2 sec, one drag every 2 sec. Cigarette smoke extract (CSE)-conditioned medium was filtered (0.22 µm) and adjusted to pH 7.2. The nicotine [(S)-3-(1-methyl-2-pyrrolidinyl)pyridine] content of GAM-CSE was determined by gas–liquid chromatography, as previously described [22] and adjusted to physiologically relevant doses [22], [23], [24], [25]. Unless otherwise noted, GAM-CSE was employed at a concentration of 4000 ng nicotine equivalents/ml. We have previously shown that *P. gingivalis* is tolerant, with respect to growth and viability, to such CSE doses [26]. In all experiments, *P. gingivalis* was either cultured in GAM or GAM-CSE for two passages. For specific experiments, CSE-exposed *P. gingivalis* was reconditioned by culturing back into GAM for two passages.

### Expression and purification of rGAPDH and truncated SspB

Recombinant GAPDH was produced by PCR amplification of the coding gene from *S. gordonii* DL-1 using primers designed using the previously published sequence for the *GAPDH* gene in *S. gordonii* [27]. To facilitate directional cloning into the expression vector system, the restriction enzyme sites SacI and XbaI (underlined) were added at the beginning of each primer for GADPH, respectively:

5′-GCGGAGCTCGTAGTTAAAGTTGGTATTAACG-3′ (forward) and

5′-GCGTCTAGACCTTTAGCGATTTTTGCG-3′ (reverse).

The PCR product obtained was initially TA-cloned into pGEM-T Easy and subsequently directionally ligated into the pBAD gIIIA expression plasmid with a C-terminal 6X-histidine tag and transformed into expression strain *E. coli* Top10. rGAPDH was induced using 2 mM arabinose and purified using HiTrap chelating HP affinity columns, according to the manufacturer's protocol. Purity was confirmed by SDS-PAGE.

### Synthesis of BAR and control peptides

BAR peptide (NH2-LEAAPKKVQDLLKKANITVKGAFQLFS-OH) and a control peptide in which BAR lysine residues were substituted with the d-enantiomers were synthesized commercially to ≥85% purity.

### Total FimA and Mfa1 expression by *P. gingivalis*

*P. gingivalis* lysates (1×10^6^ mid-late log cells) were obtained from cells grown in GAM, GAM-CSE or from reconditioned bacteria. Due to the large number of genes and proteins dysregulated by CSE [15], [28], it was necessary to normalize western blots to *P. gingivalis* cell numbers, as previously described [20], [28], [29]. Western blots were probed with either rabbit anti-FimA or anti-Mfa1 IgG and HRP-linked anti-rabbit IgG. Immunoreactive bands were visualized by chemiluminescence. Imaging and densitometry were performed using a Kodak 4000 MM image station.

### FimA and Mfa1 surface availability

FimA availability to antibody on mid- to late log phase CSE-exposed and control (GAM) *P. gingivalis* cells bound to PVC plates was determined by ELISA. Anti-FimA or anti-*P. gingivalis* IgG binding was quantified using an HRP-linked secondary antibody with tetramethylbenzidine as the chromogenic substrate, essentially as previously described by Pierce et al. [30]. Mfa1 availability to antibody was assessed identically. The FimA and Mfa1 signal was normalized to the total *P. gingivalis*. CSE-induced FimA and Mfa1 availability to antibody was expressed as percentage of the control (GAM).

### *P. gingivalis- S. gordonii* dual species biofilms

*P. gingivalis* and *S. gordonii* biofilms were formed on saliva coated cover slips in FC 71 flow cells, as we have previously described [31], [32]. Briefly, *S. gordonii* cells (25 ml cultures) were suspended in sterile PBS and labeled with hexidium iodide (1.6 mg/ml;) at room temperature for 15 min. Glass coverslips were coated with 0.22 µm filter-sterilized saliva and fixed in the flow cells. Labeled *S. gordonii* cells were then introduced into the flow cells using a Manostat Carter 4/8 cassette peristaltic pump at 6 ml per hour for approximately 2 hr. The flow cells were then washed with sterile PBS for 30 min to remove non-adherent *S. gordonii* cells. *P. gingivalis* cells, grown in GAM or GAM-CSE, were harvested by centrifugation, and re-suspended in PBS before being introduced into the flow system (flow rate of 6 ml per hour for 1 h). After washing in PBS, 30 min, bound *P. gingivalis* was detected using Alexa-488 labeled rabbit anti-*P. gingivalis* IgG. *P. gingivalis-S. gordonii* biofilms were visualized using an Olympus Fluoview confocal laser scanning microscope (Olympus, Pittsburgh, PA) under 60x magnification using the argon laser for visualization of Alexa488 labeling and the HeNe-G laser to visualize hexidium iodide-labeled streptococci. The number and height of *P. gingivalis* microcolonies were determined from 6 randomly chosen frames using the FluoView software. Microcolony depth was determined by performing *z*-plane scans from 0 µm to 50 µm above the cover glass surface.

### Induction of TNF-α and IL-6 in human innate cells by *P. gingivalis* biofilms

*P. gingivalis* biofilms were grown on pellicle coated hydroxyapatite discs (10.6 mm Ø), essentially as described previously [33]. Briefly, hydroxyapatite discs were coated for 24 hours with growth medium (60% saliva pooled from 3 different donors, 10% FBS) for 24 hrs, 37°C to allow for pellicle formation. After 24 hrs, pellicle-coated hydroxyapatite discs were immersed in GAM or CSE in 6-well polystyrene cell culture plates and seeded with 10^9^ *P. gingivalis* cells grown for two sequential passages in either GAM or CSE to initiate biofilm formation. Discs were incubated for 48 hr under anaerobic conditions (86% N_2_, 4% H_2_, 10% CO_2_) at 37°C in a Coy Laboratories anaerobic chamber. To determine the inflammatory potential of *P. gingivalis* biofilms, hydroxyapatite discs were removed from GAM/CSE, washed once in PBS before placing them in 24-well polystyrene plates containing human PBMCs (10^6^ per well). PBMCs were also simultaneously challenged with planktonic cultures of *P. gingivalis* grown in GAM or CSE for two passages (MOI 10∶1).

### Adherence of *P. gingivalis* to rGAPDH

Binding of *P. gingivalis* cells to immobilized protein was studied using a protocol adapted from Demuth et al [20]. 1, 5 and 10 µg of rGAPDH were immobilized on nitrocellulose membranes using a vacuum dot blot apparatus. Membranes were then blocked with 3% BSA for an hour followed by overnight incubation with *P. gingivalis* at 10^9^ cfu/mL from GAM/CSE cultures at RT. Bound *P. gingivalis* was then quantified using rabbit anti- *P. gingivalis* IgG followed by HRP-tagged anti-rabbit secondary antibody and DAB substrate. Mean dot intensity from three independent blots was calculated using ImageJ software (NIH) and background signals (no immobilized protein) subtracted.

### *P. gingivalis* aggregation assays

Fimbriae directly increase *P. gingivalis* adhesiveness by promoting inter-bacterial aggregation [34] [35] As CSE increases fimbrial density, we determined whether CSE promotes bacterial auto-aggregation. *P. gingivalis*, grown in GAM or CSE, was harvested by centrifugation and suspended in sterile PBS at O.D. ∼1. Aggregation of *P. gingivalis* was assessed spectrophotometerically (Abs_600__nm_) over 8 hrs using an Eppendorf Biophotometer Plus spectrophotometer.

### Influence of exogenous *S. gordonii* ligands (GAPDH and BAR) on dual species CSE-induced biofilms

CSE and control (GAM) *P. gingivalis*-*S. gordonii* biofilms were formed and analyzed, as described above with a slight modification. *P. gingivalis* grown in GAM or GAM-CSE was pre-treated with 3.4 µM GAPDH or BAR peptide for 30 minutes before introduction into the flow chamber. The number and height of *P. gingivalis* microcolonies were determined as described in the dual species biofilm section.

### Statistical Analysis

Data were evaluated by two-tailed *t* tests and analysis of variance (ANOVA), as appropriate, using the InStat program (Graphpad Software, La Jolla, CA). Statistical significance was set at ***p***<0.05.

## Results

### CSE exposure upregulates surface expression of surface-available FimA but not Mfa1

**Figure 1:**
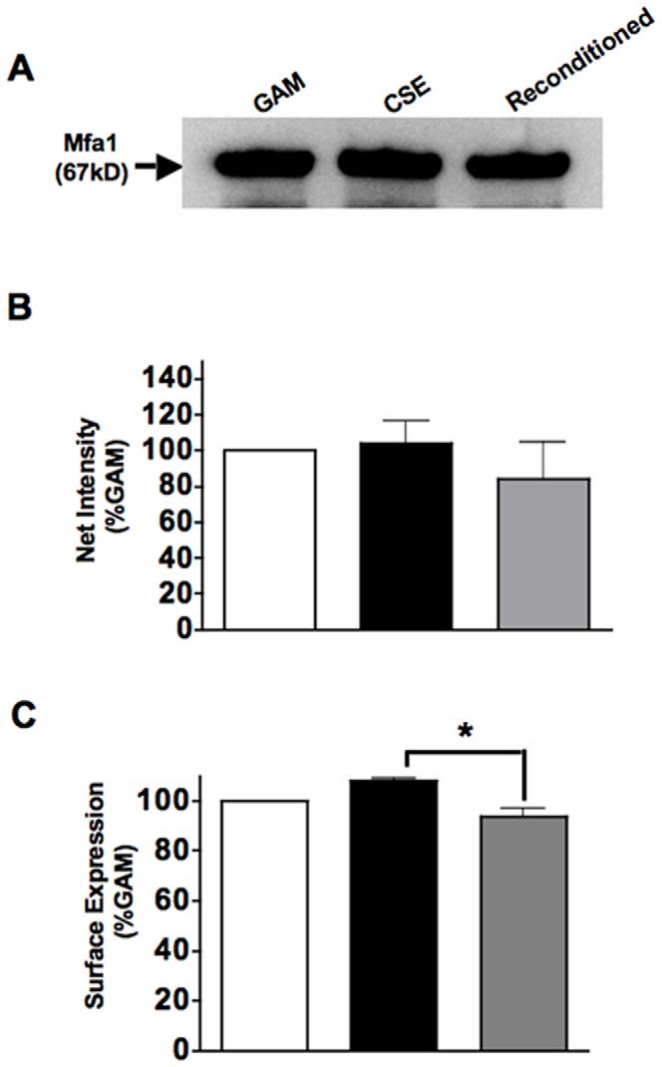
CSE exposure does not influence total protein levels or surface availability of Mfa1. (A) Western blots of 1 x 10^6^ *P. gingivalis* cell lysates sequentially passaged in GAM, GAM-CSE, and then fresh GAM, respectively and re-probed with rabbit anti-Mfa1 antibody (blot was first probed for FimA, as shown in ***Supplemental*** ***Figure 1***). (B) Relative band intensities for Mfa1 expression (B) indicate Mfa1 protein levels remain unaltered by CSE exposure. Surface availability ELISAs for Mfa1 (C) confirm that Mfa1 availability on the *P. gingivalis* surface remains unchanged. *p < 0.05.

Our previous studies suggested that exposure to CSE augments the expression of the major fimbrial antigen of *P. gingivalis* (FimA) at the transcriptional (microarray and RT-PCR) and protein levels, while simultaneously down-regulating capsular polysaccharides [15], [28]. CSE-induced upregulation of FimA is confirmed in ***Figure S1***. However, CSE exposure has no effect on the absolute protein level of the minor fimbrial antigen of *P. gingivalis* (Mfa1; ***Figure 1A*** and ***B***) or on Mfa1 surface presentation, as assessed by availability to anti-Mfa1 antibodies (***Figure 1C***).

### CSE exposure increases *P. gingivalis*-*S. gordonii* biofilm formation

**Figure 2:**
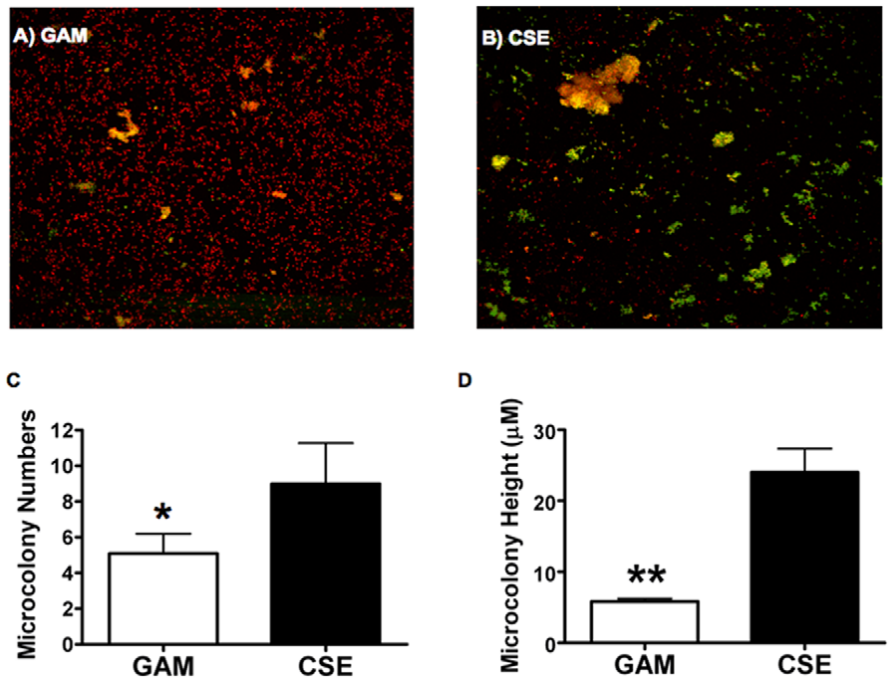
CSE exposure augments *P. gingivalis* - *S. gordonii* biofilm formation. Total number and height of microcolonies (yellow) formed by Hexidium iodide labeled *S. gordonii* (red) and Alexa-488 labeled *P. gingivalis* (green) biofilms, formed in an open flow system were quantified by taking optical section along the *x-y* and *z* axis at 1 µm intervals with a confocal microscope. CSE-exposed *P. gingivalis* associated strongly with *S. gordonii* as reflected by increased number of microcolonies formed (B) compared to control *P. gingivalis* grown in GAM (A). Total numbers (C) and average microcolony height (D) were quantified from 6 randomly chosen microscopic fields per biofilm using the FluoView software. *p<0.05; **p<0.01.

***Figures 2A*** and ***2B*** show representative z-stack images of microcolonies formed by *P. gingivalis* grown in GAM and GAM-CSE, respectively, on *S. gordonii*. As presented in ***Figure 2C and 2D***, CSE exposure resulted in an approximately 2-fold increase in the total number of *P. gingivalis*-*S. gordonii* microcolonies formed as well as an approximately 3-fold increase in microcolony height, relative to control biofilms (p<0.05 and p<0.01, respectively).

### CSE exposure decreases the inflammatory activity of *P. gingivalis* biofilms

**Figure 3:**
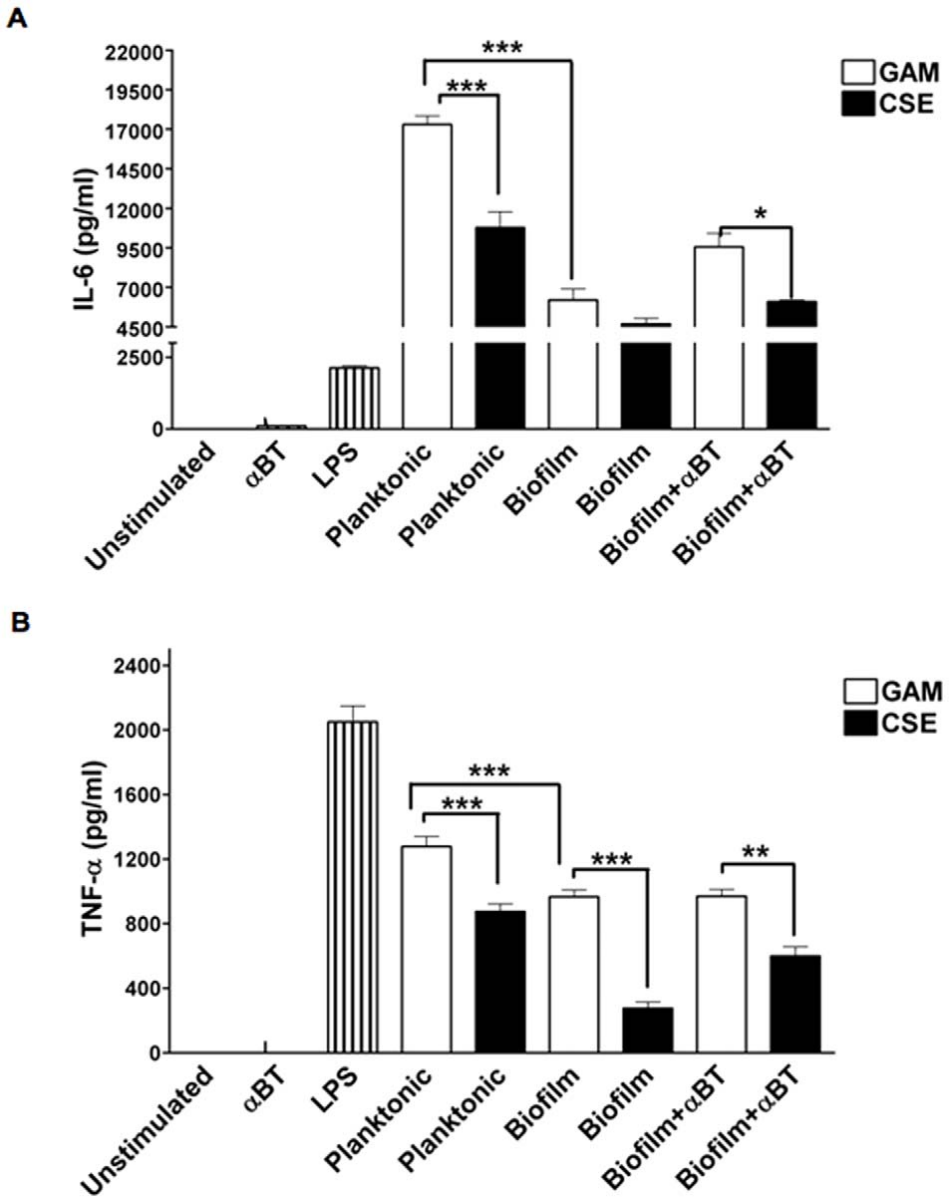
CSE exposure reduces inflammatory activity of *P. gingivalis* biofilms. Human PBMCs (1×10^6^) were challenged with *P. gingivalis* grown in GAM or CSE as planktonic cultures (MOI 10∶1) or with 48 hr *P. gingivalis* biofilms formed on HA discs in GAM or CSE and with 1 µg/ml *E. coli* LPS as positive control. Additionally, PBMCs were pre-treated with α–bungarotoxin (500 ng/ml for 30 min) before addition of biofilm discs. Cells were stimulated overnight and cell-free supernatants were analyzed for IL-6 (A) and TNF-α (B) levels by ELISA. *p<0.05; **p<0.01; ***p<0.001.

The pro-inflammatory activity of CSE-exposed *P. gingivalis* (Black bars) grown either in planktonic form or in monospecies biofilms was lower than that of unexposed bacteria (white bars), as determined by IL-6 and TNF-α release from primary human PBMCs, and- shown in ***Figure 3A and B*** respectively. Furthermore, this reduced immune potential was not influenced by á-bungarotoxin, an á7 nAChR and cholinergic anti-inflammatory pathway antagonist [36], (see ***Figure 3***). Therefore, it is unlikely that repressed cytokine production can be attributed to nicotine carry over from growth medium or bacterial sequestration and, rather, is a direct consequence of CSE-induced alteration to *P. gingivalis* itself.

### CSE exposure does not induce *P. gingivalis* auto-aggregation

Aggregation of planktonic *P. gingivalis* did not occur in *P. gingivalis* grown in CSE or in GAM-CSE (see ***Figure S2***). Rather, the slow settling that is typical of this bacterium was apparent, suggesting that auto-aggregation does not contribute to CSE-induced *P. gingivalis*-*S. gordonii* biofilm formation.

### CSE exposure increases *P. gingivalis* binding to immobilized FimA ligand (rGAPDH)

**Figure 4:**
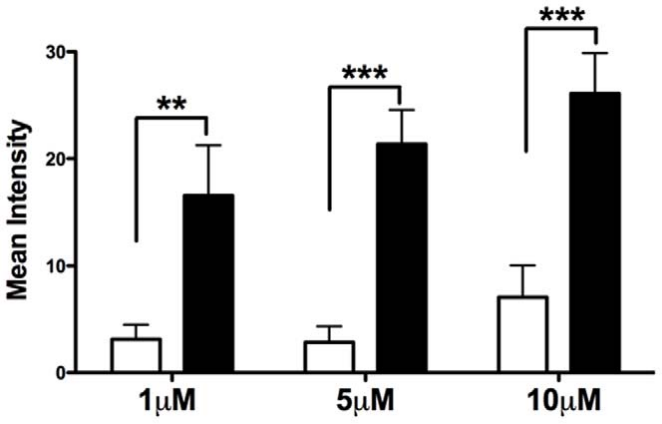
CSE exposure promotes adherence of intact *P. gingivalis* to rGAPDH. *P. gingivalis* adherence to immobilized rGAPDH was assessed by dot blot. 10^9^ cfu/ml of *P. gingivalis*, grown either in GAM or CSE, were added to each blot and adherent bacteria detected using anti-*P. gingivalis* IgG. Averaged mean intensities from three independent blots indicate CSE exposure augments binding to GAPDH in a dose-dependent manner. **p<0.01; ***p<0.001.

To determine whether CSE-induced FimA upregulation played a functional role in *P. gingivalis* - *S. gordonii* associations, adherence of *P. gingivalis* to immobilized rGAPDH was assessed. As shown in **Figure 4**, CSE-exposed *P. gingivalis* exhibited increased binding to rGAPDH (p<0.01 and p<0.001), compared to control bacteria (GAM), and did so in a dose-dependent manner.

### CSE-induced *P. gingivalis-S. gordonii* biofilm formation is FimA-enhanced but Mfa1-dependent

**Figure 5:**
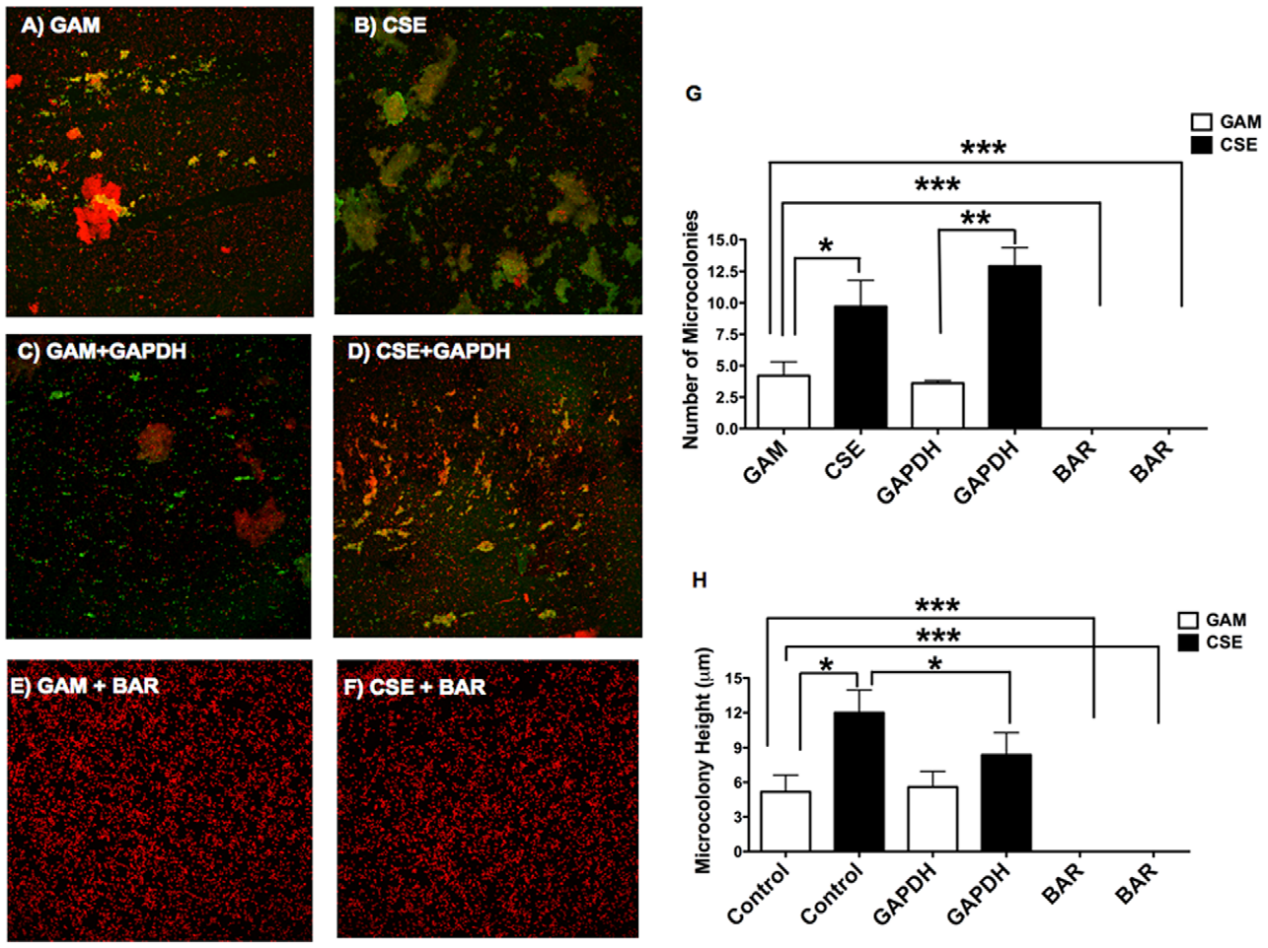
CSE augmentation of biofilm formation is FimA-enhanced and Mfa1-dependent. As shown previously CSE exposure significantly increased *P. gingivalis* association with *S. gordonii* (B) compared to control *P. gingivalis* grown in GAM (A) as reflected by total numbers of microcolonies (yellow) formed. Pre-treatment of *P. gingivalis* with rGAPDH (3.4 µM) reduced association (microcolony height and numbers) between CSE-exposed *P. gingivalis* and *S. gordonii* (D) but had no effect on control *P. gingivalis* grown in GAM (C). Pretreatment with BAR peptide (3.4 µM) completely abolished any association between *S. gordonii* and *P. gingivalis* grown either in GAM (E) or CSE (F). Total number and height of microcolonies (yellow) formed by Hexidium iodide labeled *S. gordonii* (red) and Alexa488 labeled *P. gingivalis* (green) biofilms formed in an open flow system were quantified by taking optical section along the *x-y* and *z* axis at 1 µm intervals under a confocal microscope and presented in figures (G) and (H) respectively. *p<0.05; **p<0.01; ***p<0.001.

The efficacy of rGAPDH protein to interfere with CSE-induced *P. gingivalis-S. gordonii* biofilms formation was subsequently assessed. CSE-exposed *P. gingivalis* associated strongly with *S. gordonii*, compared to controls (***Figures 5A*** and ***B***). While exogenous rGAPDH pretreatment did not have a significant influence on microcolony numbers, rGAPDH did decrease microcolony height by 65% relative to control biofilms (p<0.05 and p<0.01); (***Figures*** ** ***5C and D***). We have previously shown that a synthetic BAR peptide, characterized by Daep et al, efficiently blocks the binding of Mfa1 to SspB [32], [37]. Therefore, we used pre-incubation with BAR to ascertain the importance of Mfa1 in CSE-induced *P. gingivalis-S. gordonii* biofilm formation. As shown in ***Figure 5E*** and ***5F***, BAR peptide at 3.4 µM completely abrogated (100%) dual species biofilm formation under CSE-exposed and control conditions.

## Discussion

Our prior work has established that CSE-exposure increases *P. gingivalis* monospecies biofilm formation. However, *in vivo* it is *P. gingivalis*-early colonizer (particularly streptococcal) interactions that are considered critical for *P. gingivalis* to establish itself and maintain a presence in dental plaque. Furthermore, microbes may interact with the immune response in different manners in biofilms than in planktonic form. Thus we have extended our prior studies to observe the influence of CSE on *P. gingivalis* – *S. gordonii*; mechanisms that promote CSE-induced *P. gingivalis* – *S. gordonii* interaction; and the pro-inflammatory potential of CSE-exposed biofilms.

We have previously reported, using microarrays representative of the *P. gingivalis* genome that CSE exposure resulted in the differential regulation of 6.8% of *P. gingivalis* genes in planktonic culture. Of particular relevance to biofilm formation were two major fimbriae synthesis genes that were induced by CSE (PG2133 and PG2134) as well as several genes in the capsular operon (*cap*K [PG0111], PG0117, PG0118 and *wec*C [PG0108]) that were suppressed by CSE [15]. CSE dysregulation of capsular and fimbrial genes was reflected at the ultrastructural level, as confirmed by electron microcopy, and at the functional level, as shown by altered host-pathogen interactions [15], [28].

We now show that CSE-exposure results in increased surface availability of FimA protein, but not Mfa1, and increases *P. gingivalis* biofilm formation in a dual species model. Thus, CSE-induced fimbrial gene dysregulation is functionally significant. FimA is known to play multiple roles in *P. gingivalis* virulence, but is best characterized as a fimbrial adhesin that is important in binding of *P. gingivalis* to the salivary pellicle [38].

Mfa1 is essential for *P. gingivalis*-*S. gordonii* biofilm formation, as illustrated by SspB-deficient mutants and competitive inhibition with BAR peptide [19], [31], [32], [39]. We show herein that, nevertheless, FimA binds strongly to immobilized rGADPH; CSE-induced FimA surface upregulation significantly enhances dual species biofilm; and that the addition of an exogenous FimA ligand suppresses *P. gingivalis* - *S. gordonii* biofilms. FimA-GAPDH interactions are of much lower affinity than Mfa1-SSpB/BAR interactions, with IC_50_ values for inhibition of dual species biofilms reported to be in the region of 300-400 µg/ml and 4 µg/ml for GAPDH and SspB peptides respectively [31], [32], [40]. Thus, it would seem that a CSE-induced increase in FimA avidity enhances initial *P. gingivalis*-*S. gordonii* adhesive contacts. Subsequently, high affinity Mfa-SspB interactions are critical in stabilizing the biofilm and possibly promoting *P. gingivalis* persistence in the host.

While auto-aggregation in planktonic culture may not be directly related to biofilm formation *in vivo*, the lack of CSE-induced *P. gingivalis* auto-auggregation noted in vitro suggests that bacterial clumping did not contribute to increased biofilm formation in the flow cell system.

These data suggest that, in smokers and non-smokers alike, anti-biofilm strategies that target both FimA and Mfa1 may be more successful in inhibiting *P. gingivalis* colonization than the use of anti-Mfa1 agents alone. They also suggest that the considerable efforts currently underway to develop FimA-focused vaccination strategies [41], [42] may be particularly relevant to tobacco users, the section of society who are most likely to harbor *P. gingivalis* and who share the largest periodontal disease burden. This hypothesis is supported by the discovery of a FimA binding domain (amino acid residues 166 to 183) on the GADPH protein produced by another oral streptococcus species, *S. oralis*, along with the realization that this peptide may represent another potent inhibitor of *P. gingivalis* colonization in the oral cavity [40].

It should also be remembered that enhancement of FimA surface expression may have other pathological consequences that are unrelated to streptococcal adhesion. Recently, for example, it has been shown that FimA binds several plasma factors, particularly prekallikrein, and may thus be involved in subversion of the kinin generating system to the benefit of *P. gingivali*s and disadvantage of the host [43]. FimA also appears to play important roles in the invasion and activation of host cells such as osteoblasts [44] and vascular endothelial cells [45] by *P. gingivalis*. Indeed, the invasion and activation of host cells is expected to be a critical step in many of the fatal and debilitating chronic systemic diseases associated with periodontal infections. Enhanced FimA expression may also promote *P. gingivalis* adherence to other periodontal pathogens like *Treponema denticola* [46] promoting establishment of a complex pathogenic plaque.

It is of interest that initial characterization of the host response to CSE-enhanced *P. gingivalis* mono-species biofilms suggests that they exhibit considerably reduced inflammatory potential than control biofilms, as assessed by stimulation of TNF-á and IL-6 from primary human innate cells. *P. gingivalis* does not express nicotinic receptors, does not internalize nicotine [47], [48] and bacteria were washed prior to stimulating PBMCs with biofilms. Nevertheless, we thought it prudent to ensure that inflammatory suppression subsequent to engagement of the á7 nicotinic acetylcholine receptor by nicotine [36] did not occur. The reduced cytokine profile observed with CSE-exposed biofilms was not influenced by pre-incubation with the nAChR antagonist, á-bungarotoxin. Therefore, the possibility of nAChR-initiated activation of the cholinergic anti-inflammatory pathway by nicotine carry over is unlikely. These results are in keeping with our prior in vitro studies that have shown that intact *P. gingivalis* exposed to CSE elicit a reduced ability to induce cytokines under the transcriptional control of the NF-êB system [15], [28]. While CSE promotes FimA expression, FimA itself is an inefficient TLR2-agonist and induces immune hyporesponsiveness in innate cells in a TLR2-IRAK-1-IκBα-dependent manner [28]. More recent data suggest that the TLR-engagement domain of FimA lies in the signal peptide with a cysteine residue at single amino acid position 19A essential [49]. Our findings are also consistent with established *in vivo* conditions. Patients who smoke exhibit increased susceptibility to periodontitis and are more likely to display severe disease and be refractory to treatment [5]. Paradoxically, smokers demonstrate reduced clinical inflammation, that is, diminished edema, bleeding on probing, and local cytokine production compared to non-smokers [5], [50]. Thus, a reduced inflammatory capacity of CSE-exposed *P. gingivalis* biofilms is in keeping with the established clinical apparition of periodontitis in tobacco smokers.

In summary, the majority of periodontitis cases in the US have been directly attributed to tobacco smoking [6], yet the mechanisms underlying this smoking-induced and exacerbated infectious disease are poorly understood. CSE exposure results in increased dual species biofilm formation in a manner that is FimA-enhanced, Mfa1-dependent, and does not involve CSE-induced *P. gingivalis* aggregation. The dramatic CSE-induced promotion of biofilm formation is likely to contribute to the established clinical phenomenon of increased *P. gingivalis* infection and perseverance in the oral cavity of smokers. These findings are also relevant to ongoing efforts to develop effective anti-FimA vaccination strategies for the treatment of periodontal diseases and may have implications for other biofilm-associated diseases.

## Supporting Information

Figure S1 **CSE exposure increases total protein levels and surface availability of FimA.** (A) Western blots of 1×10^6^ *P. gingivalis* cell lysates sequentially passaged in GAM, GAM-CSE, and then fresh GAM, respectively and probed with rabbit anti-FimA antibody (A). (B) Relative band intensities for FimA expression indicate FimA is reversibly and significantly upregulated on CSE-exposure. Surface availability ELISA for FimA confirms that upregulated FimA is available on the *P. gingivalis* surface. *p<0.05; **p<0.01; ***p<0.001.
(TIF)

Figure S2 **CSE does not induce** ***P. gingivalis*** **auto-aggregation.** Aggregation of *P. gingivalis* (10^9^ cells/ml) grown in either in GAM (grey line) or CSE (black line) and suspended in PBS was monitored by measuring optical density (O.D._600_) of over time. CSE does not increase *P. gingivalis* aggregation.
(TIFF)

## References

[1] Grossi SG, Genco RJ, Machtei EE, Ho AW, Koch G (1995). Assessment of risk for periodontal disease. II. Risk indicators for alveolar bone loss.. Journal of Periodontology.

[2] Grossi SG, Zambon JJ, Ho AW, Koch G, Dunford RG (1994). Assessment of risk for periodontal disease. I. Risk indicators for attachment loss.. J Periodontol.

[3] McGuire MK, Nunn ME (1996). Prognosis versus actual outcome. III. The effectiveness of clinical parameters in accurately predicting tooth survival.. J Periodontol.

[4] Bagaitkar J, Demuth DR, Scott DA (2008). Increased susceptibility to bacterial infections in tobacco smokers.. Tobacco Induced Diseases.

[5] Palmer RM, Wilson RF, Hasan AS, Scott DA (2005). Mechanisms of action of environmental factors–tobacco smoking.. J Clin Periodontol.

[6] Tomar SL, Asma S (2000). Smoking-attributable periodontitis in the United States: findings from NHANES III. National Health and Nutrition Examination Survey.. J Periodontol.

[7] Brown LJ, Johns BA, Wall TP (2002). The economics of periodontal diseases.. Periodontol 2000.

[8] Rosan B, Lamont RJ (2000). Dental plaque formation.. Microbes Infect.

[9] Marsh PD (1994). Microbial ecology of dental plaque and its significance in health and disease.. Adv Dent Res.

[10] Christopher AB, Arndt A, Cugini C, Davey ME (2010). A streptococcal effector protein that inhibits Porphyromonas gingivalis biofilm development.. Microbiology.

[11] Haffajee AD, Socransky SS (2001). Relationship of cigarette smoking to the subgingival microbiota.. J Clin Periodontol.

[12] Zambon JJ, Grossi SG, Machtei EE, Ho AW, Dunford R (1996). Cigarette smoking increases the risk for subgingival infection with periodontal pathogens.. J Periodontol.

[13] Eggert FM, McLeod MH, Flowerdew G (2001). Effects of smoking and treatment status on periodontal bacteria: evidence that smoking influences control of periodontal bacteria at the mucosal surface of the gingival crevice.. Journal of Periodontology.

[14] Kamma JJ, Nakou M, Baehni PC (1999). Clinical and microbiological characteristics of smokers with early onset periodontitis.. J Periodontal Res.

[15] Bagaitkar J, Scott DA, Williams LR, Renaud DE, Daep C (2009). Tobacco-induced alterations to Porphyromonas gingivalis-host interactions.. Environmental Microbiology.

[16] Chung WO, Demuth DR, Lamont RJ (2000). Identification of a Porphyromonas gingivalis receptor for the Streptococcus gordonii SspB protein.. Infect Immun.

[17] Maeda K, Nagata H, Yamamoto Y, Tanaka M, Tanaka J (2004). Glyceraldehyde-3-phosphate dehydrogenase of Streptococcus oralis functions as a coadhesin for Porphyromonas gingivalis major fimbriae.. Infect Immun.

[18] Maeda K, Nagata H, Nonaka A, Kataoka K, Tanaka M (2004). Oral streptococcal glyceraldehyde-3-phosphate dehydrogenase mediates interaction with Porphyromonas gingivalis fimbriae.. Microbes Infect.

[19] Park Y, Simionato MR, Sekiya K, Murakami Y, James D (2005). Short fimbriae of Porphyromonas gingivalis and their role in coadhesion with Streptococcus gordonii.. Infect Immun.

[20] Demuth DR, Irvine DC, Costerton JW, Cook GS, Lamont RJ (2001). Discrete protein determinant directs the species-specific adherence of Porphyromonas gingivalis to oral streptococci.. Infect Immun.

[21] Davey ME, Duncan MJ (2006). Enhanced biofilm formation and loss of capsule synthesis: deletion of a putative glycosyltransferase in Porphyromonas gingivalis.. J Bacteriol.

[22] Fraser HS, Palmer RM, Wilson RF, Coward PY, Scott DA (2001). Elevated systemic concentrations of soluble ICAM-1 (sICAM) are not reflected in the gingival crevicular fluid of smokers with periodontitis.. J Dent Res.

[23] McGuire JR, McQuade MJ, Rossmann JA, Garnick JJ, Sutherland DE (1989). Cotinine in saliva and gingival crevicular fluid of smokers with periodontal disease.. J Periodontol.

[24] Chen X, Wolff L, Aeppli D, Guo Z, Luan W (2001). Cigarette smoking, salivary/gingival crevicular fluid cotinine and periodontal status. A 10-year longitudinal study.. J Clin Periodontol.

[25] Scott DA, Palmer RM, Stapleton JA (2001). Validation of smoking status in clinical research into inflammatory periodontal disease.. J Clin Periodontol.

[26] Bagaitkar J, Williams LR, Renaud DE, Bemakanakere MR, Martin M (2009). Tobacco-induced alterations to Porphyromonas gingivalis-host interactions.. Environ Microbiol.

[27] Demuth DR, Davis CA, Corner AM, Lamont RJ, Leboy PS (1988). Cloning and expression of a Streptococcus sanguis surface antigen that interacts with a human salivary agglutinin.. Infect Immun.

[28] Bagaitkar J, Demuth DR, Daep CA, Renaud DE, Pierce DL (2010). Tobacco upregulates P. gingivalis fimbrial proteins which induce TLR2 hyposensitivity.. PLoS One.

[29] Hritz M, Fisher E, Demuth DR (1996). Differential regulation of the leukotoxin operon in highly leukotoxic and minimally leukotoxic strains of Actinobacillus actinomycetemcomitans.. Infect Immun.

[30] Pierce DL, Nishiyama S, Liang S, Wang M, Triantafilou M (2009). Host adhesive activities and virulence of novel fimbrial proteins of Porphyromonas gingivalis.. Infect Immun.

[31] Lamont RJ, El-Sabaeny A, Park Y, Cook GS, Costerton JW (2002). Role of the Streptococcus gordonii SspB protein in the development of Porphyromonas gingivalis biofilms on streptococcal substrates.. Microbiology.

[32] Daep CA, James DM, Lamont RJ, Demuth DR (2006). Structural characterization of peptide-mediated inhibition of Porphyromonas gingivalis biofilm formation.. Infect Immun.

[33] Guggenheim B, Stathopolou P, Thurheer T, Kinane D (2009). In vitro modeling of host-parasite interactions: the ‘subgingival’ biofilm challenge of primary human epithelial cells.. Nature Immunology - Technical Reports. In press.

[34] Hongo H, Takano H, Morita M (2007). Dense fimbrial meshwork enhances Porphyromonas gingivalis adhesiveness: a scanning electron microscopic study.. J Periodontal Res.

[35] Kuboniwa M, Amano A, Hashino E, Yamamoto Y, Inaba H (2009). Distinct roles of long/short fimbriae and gingipains in homotypic biofilm development by Porphyromonas gingivalis.. BMC Microbiol.

[36] Rehani K, Scott DA, Renaud D, Hamza H, Williams LR (2008). Cotinine-induced convergence of the cholinergic and PI3 kinase-dependent anti-inflammatory pathways in innate immune cells.. Biochim Biophys Acta.

[37] Daep CA, Lamont RJ, Demuth DR (2008). Interaction of Porphyromonas gingivalis with oral streptococci requires a motif that resembles the eukaryotic nuclear receptor box protein-protein interaction domain.. Infection and Immunity.

[38] Koh EM, Kim J, Kim TG, Moon JH, Oh JH (2011). Cloning and characterization of heavy and light chain genes encoding the FimA-specific monoclonal antibodies that inhibit Porphyromonas gingivalis adhesion.. Microbiol Immunol.

[39] Daep CA, Lamont RJ, Demuth DR (2008). Interaction of Porphyromonas gingivalis with oral streptococci requires a motif that resembles the eukaryotic nuclear receptor box protein-protein interaction domain.. Infect Immun.

[40] Nagata H, Iwasaki M, Maeda K, Kuboniwa M, Hashino E (2009). Identification of the binding domain of Streptococcus oralis glyceraldehyde-3-phosphate dehydrogenase for Porphyromonas gingivalis major fimbriae.. Infect Immun.

[41] Shin EA, Park YK, Lee KO, Langridge WH, Lee JY (2009). Synthesis and assembly of Porphyromonas gingivalis fimbrial protein in potato tissues.. Mol Biotechnol.

[42] Yu F, Xu QA, Chen W (2011). A targeted fimA DNA vaccine prevents alveolar bone loss in mice after intra-nasal administration.. J Clin Periodontol.

[43] Rapala-Kozik M, Bras G, Chruscicka B, Karkowska-Kuleta J, Sroka A (2011). Adsorption of components of the plasma kinin-forming system on the surface of Porphyromonas gingivalis involves gingipains as the major docking platforms.. Infect Immun.

[44] Zhang W, Ju J, Rigney T, Tribble GD (2011). Fimbriae of Porphyromonas gingivalis are Important for Initial Invasion of Osteoblasts, but Not for Inhibition of Their Differentiation and Mineralization.. J Periodontol.

[45] Khlgatian M, Nassar H, Chou HH, Gibson FC, 3rd, Genco CA (2002). Fimbria-dependent activation of cell adhesion molecule expression in Porphyromonas gingivalis-infected endothelial cells.. Infect Immun.

[46] Hashimoto M, Ogawa S, Asai Y, Takai Y, Ogawa T (2003). Binding of Porphyromonas gingivalis fimbriae to Treponema denticola dentilisin.. FEMS Microbiol Lett.

[47] Cogo K, Calvi BM, Mariano FS, Franco GC, Goncalves RB (2009). The effects of nicotine and cotinine on Porphyromonas gingivalis colonisation of epithelial cells.. Arch Oral Biol.

[48] Cogo K, Montan MF, Bergamaschi Cde C, E DA, Rosalen PL (2008). In vitro evaluation of the effect of nicotine, cotinine, and caffeine on oral microorganisms.. Can J Microbiol.

[49] Shoji M, Yoshimura A, Yoshioka H, Takade A, Takuma Y (2010). Recombinant Porphyromonas gingivalis FimA preproprotein expressed in Escherichia coli is lipidated and the mature or processed recombinant FimA protein forms a short filament in vitro.. Can J Microbiol.

[50] Johnson GK, Guthmiller JM (2007). The impact of cigarette smoking on periodontal disease and treatment.. Periodontol 2000.

