# Supplementary figures and images for: Tobacco Smoke Augments Porphyromonas gingivalis - Streptococcus gordonii Biofilm Formation
Source: PLoS One. 2011 Nov 14;6(11):e27386. doi: 10.1371/journal.pone.0027386 (PMC3215692; doi:10.1371/journal.pone.0027386)

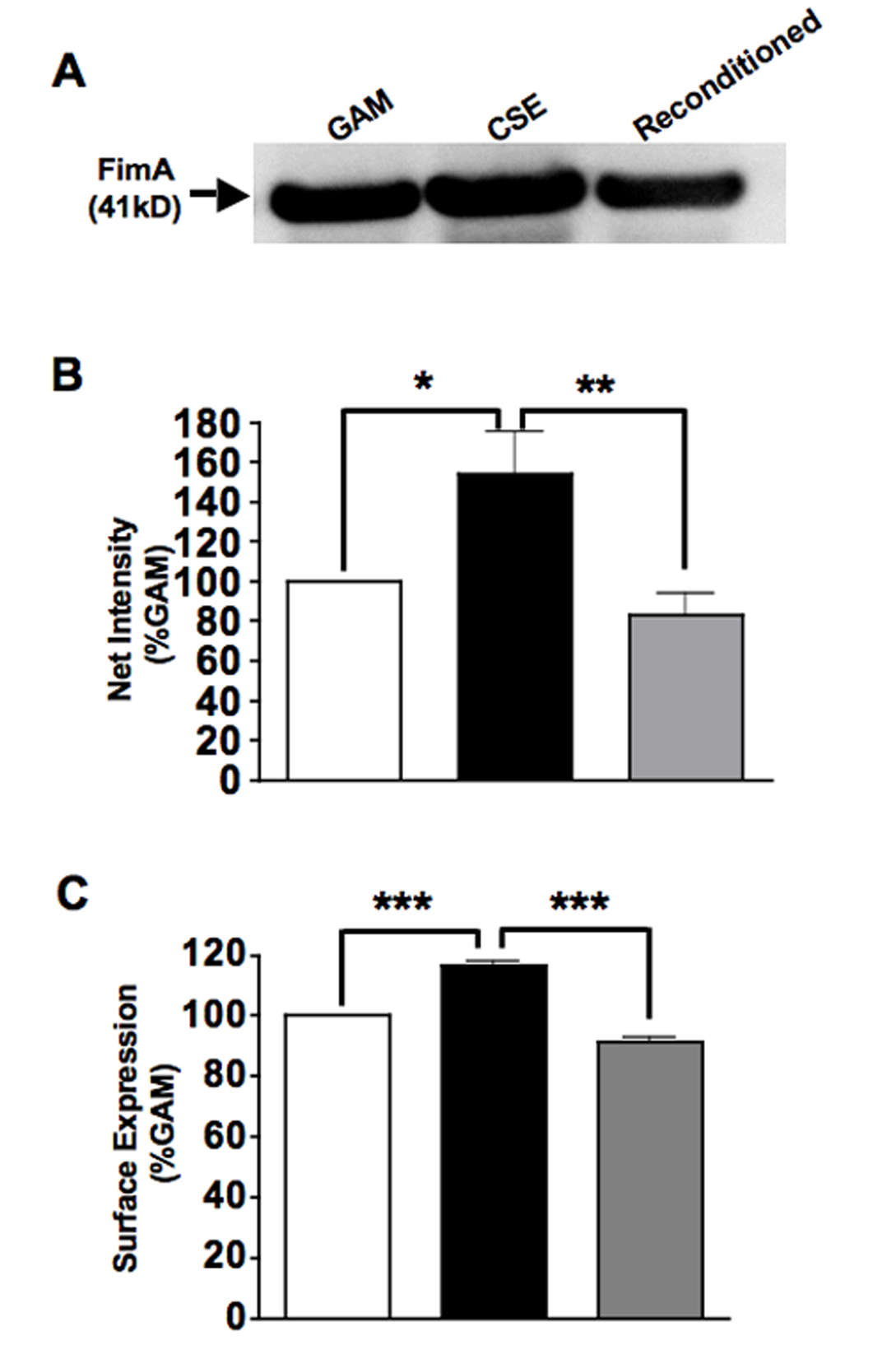

Supplement: Figure S1 — CSE exposure increases total protein levels and surface availability of FimA. (A) Western blots of 1×10^6^ P. gingivalis cell lysates sequentially passaged in GAM, GAM-CSE, and then fresh GAM, respectively and probed with rabbit anti-FimA antibody (A). (B) Relative band intensities for FimA expression indicate FimA is reversibly and significantly upregulated on CSE-exposure. Surface availability ELISA for FimA confirms that upregulated FimA is available on the P. gingivalis surface. *p<0.05; **p<0.01; ***p<0.001. (TIF) [file pone.0027386.s001.tif]

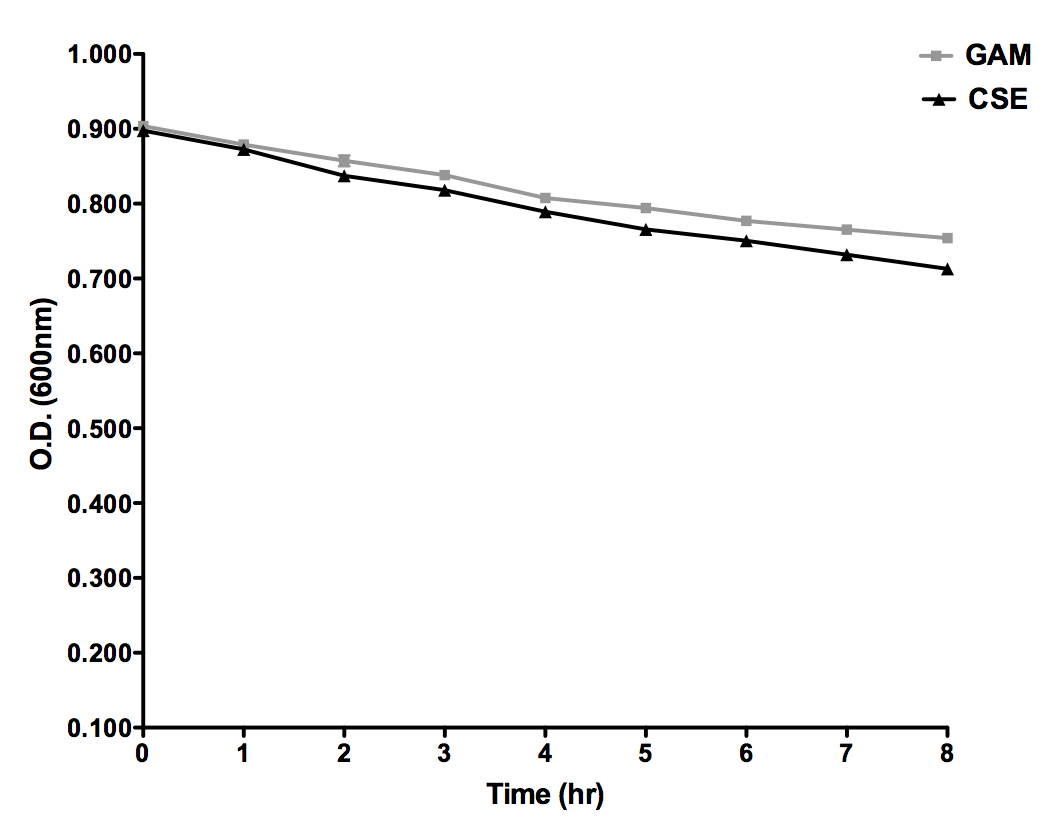

Supplement: Figure S2 — CSE does not induce P. gingivalis auto-aggregation. Aggregation of P. gingivalis (109 cells/ml) grown in either in GAM (grey line) or CSE (black line) and suspended in PBS was monitored by measuring optical density (O.D.600) of over time. CSE does not increase P. gingivalis aggregation. (TIFF) [file pone.0027386.s002.tiff]
